# Hypoxia-related Y RNA fragments as a novel potential biomarker for distinguishing metastatic oral melanoma from non-metastatic oral melanoma in dogs

**DOI:** 10.1080/01652176.2023.2300943

**Published:** 2024-01-30

**Authors:** MD Nazmul Hasan, MD Mahfuzur Rahman, Al Asmaul Husna, Daiki Kato, Takayuki Nakagawa, Mohammad Arif, Naoki Miura

**Affiliations:** a Joint Graduate School of Veterinary Medicine, Kagoshima University, Korimoto, Kagoshima, Japan; b Veterinary Teaching Hospital, Joint Faculty of Veterinary Medicine, Kagoshima University, Korimoto, Kagoshima, Kagoshima, Japan; c Department of Human Oncology, University of WI School of Medicine and Public Health, Madison, WI, USA; d Laboratory of Veterinary Surgery, Graduate School of Agricultural and Life Sciences, The University of Tokyo, Bunkyo, Tokyo, Japan

**Keywords:** Hypoxia, Y RNA, dog, melanoma, next-generation sequencing

## Abstract

Hypoxia may promote tumor progression, and hypoxically altered noncoding RNA (ncRNA) expression may play a role in metastasis. Canine oral melanoma (COM) frequently metastasizes, and ncRNA expression under hypoxia may be clinically significant. We aimed to elucidate ncRNA fragments whose expression is altered by hypoxia in COM-derived primary KMeC and metastatic LMeC cell lines using next-generation sequencing to validate these results in qRT-PCR, and then compare expression between metastatic and non-metastatic COM. The NGS analysis and subsequent qRT-PCR validation were performed using hypoxic and normoxic KMeC and LMeC cells, and clinical samples [tumor tissue, plasma, and plasma-derived extracellular vesicles] obtained from dogs with metastatic or non-metastatic melanoma were analyzed with qRT-PCR. Y RNA was significantly decreased in metastatic LMeC cells versus primary KMeC cells in hypoxic and normoxic conditions. The expression of Y RNA was decreased in dogs with metastatic melanoma versus those with non-metastatic melanoma for all clinical sample types, reflecting the pattern found with hypoxia. Receiver operating characteristic analysis demonstrated that Y RNA level is a promising biomarker for discriminating metastatic from non-metastatic melanoma in plasma [area under the curve (AUC) = 0.993, *p* < 0.0001] and plasma-derived extracellular vesicles (AUC = 0.981, *p* = 0.0002). Overall, Y RNA may be more resistant to hypoxic stress in the metastatic than the non-metastatic state for COM. However, further investigation is required to elucidate the biological functions of Y RNA under hypoxic conditions.

## 1. Introduction

Hypoxia frequently develops in solid tumors as their abnormal growth and disrupted blood flow render them susceptible to oxygen shortage (Emami Nejad et al. 2021). Hypoxia can become a key characteristic of the tumor microenvironment and promote the development of metastatic tumors (Rankin and Giaccia 2016). As tumor cells mount a response to hypoxia, altered RNA expression may occur. Hypoxia-induced non-coding RNAs (ncRNAs) act as promotors of hypoxia-driven cancer progression, altering gene expression at transcriptional and post-transcriptional levels (Barreca et al. 2021). Given the role of hypoxia in promoting metastasis, determining ncRNAs whose expression is altered under hypoxia may identify potential diagnostic and prognostic biomarkers in oncology.

Canine oral melanoma (COM) may provide a fruitful line of research for hypoxia-related dysregulation. Oral melanoma is a frequent and highly aggressive cancer in humans and dogs, and the canine disease has a high metastatic propensity (Iussich et al. 2017; Turek et al. 2020; Hardwick 2021). In dogs, malignant melanoma has reported fatality rates of up to 75%, and death may often prove unpreventable even with 1-year treatment involving surgery and subsequent adjuvant therapy (Treggiari et al. 2016; Iussich et al. 2017). Dogs may serve as a model for research on human melanoma, owing to their phylogenetic proximity to humans (Palma et al. 2021; Pinto et al. 2023). Thus, investigating hypoxia-related changes in ncRNA expression may assist in the identification of promising biomarkers for canine and, ultimately, human patients. Among the ncRNAs, Y RNAs are the least investigated RNA species in COM.

Our laboratory has previously reported on hypoxia-related miRNAs in COM progression (Hino et al. 2021). Our techniques involve initially identifying small ncRNAs through next-generation sequencing (NGS) with primary and metastatic cell lines. We then validate the NGS results in qRT-PCR and perform further investigation with clinical samples from dogs with COM. The clinical samples can include tissue samples from the melanoma site and plasma, and extracellular vesicles (EVs).

EVs play a crucial role in the hypoxic tumor microenvironment (Bister et al. 2020). They act as a ‘cargo carrier’ and can release numerous dysregulated ncRNAs in several human cancer diseases, a role they also play in COM (Lunavat et al. 2015; Li et al. 2022).

Accordingly, we aimed to identify hypoxia-related ncRNAs other than miRNAs that may have potential as diagnostic biomarkers for COM.

## 2. Materials and methods

### 2.1. Experimental design

This study involved identifying hypoxically aberrantly expressed ncRNAs in primary and metastatic melanoma cell lines using NGS. The expression of the selected ncRNA was then validated in metastatic and non-metastatic melanoma clinical tissue, plasma, and plasma EV samples, to investigate differentiation between metastatic and non-metastatic melanoma.

### 2.2. Clinical sample population

Clinical samples were selected from a pool of COM samples established by the Kagoshima University Veterinary Teaching Hospital (KUVTH; Kagoshima, Japan). The samples were obtained from dogs undergoing surgery for melanoma at KUVTH or an affiliated local clinic. Written informed consent was obtained from the owner of each patient. The present study was approved by the institutional animal care ethical committee of KUVTH (Approval No. KVH220001). A certified veterinary pathologist at KUVTH confirmed the diagnosis of melanoma for each patient. Tissue samples and blood samples were processed for RNA isolation and stored at −80 °C freezer for further downstream analysis as described previously (Rahman et al. 2020).

### 2.3. Cell lines and cell culture

In this study, we used two canine oral melanoma (COM)-derived cell lines, the KMeC and LMeC cell lines (biological replicates/line:6), which originated from primary and metastatic sites, respectively (Inoue et al. 2004). We previously described hypoxic and normoxic cell preparation (Hino et al. 2021). Cells were counted using an automated cell counter (LUNAII, Logos Biosystems).

### 2.4. Isolation of EVs

EVs were isolated from plasma samples using the Total Exosome RNA and Protein Isolation Kit (Invitrogen, Thermo Fisher Scientific). First, a 300-μl aliquot from each plasma sample was mixed with a half volume of 1× PBS. A 90 μl amount of exosome precipitation reagent was then added, and the samples were subject to thorough vortex mixing and centrifuged at 10,000×*g* for 5 min. The supernatant was aspirated, and the pellet was resuspended using 150 μl 1× PBS and kept at −80 °C for downstream analysis.

### 2.5. RNA extraction

Total RNA was isolated from hypoxic and normoxic cells and tissues using a mirVana™ RNA Isolation Kit (Thermo Fisher Scientific), and from plasma samples and EVs using a mirVana PARIS Kit (Thermo Fisher Scientific, Waltham, MA, USA), in accordance with the manufacturer’s instructions for the relevant kit. To normalize differences before extraction, each plasma or plasma-derived EV sample was mixed with 5 μl of synthetic cel-miR-39. RNA quality and integrity were evaluated using an Agilent 2100 Bioanalyzer (G2939BA, Agilent Technologies, Santa Clara, CA, USA). The RNA Integrity Numbers (RINs) for both tumor tissues and cell samples ranged from 8.5 to 9.5.

### 2.6. Next-generation sequencing

Total RNA sequencing was performed at Hokkaido System Science, Co. Ltd., Sapporo, Japan. The procedures were described previously (Hino et al. 2021). In brief, small RNA libraries were built based on a 1 µg amount of total RNA using the TruSeq Small RNA Library Preparation Kit (Illumina, San Diego, California) in accordance with the kit instructions. Following library preparation, adapters (5′ and 3′) were ligated to the small RNAs, and cDNA was generated via reverse transcription and amplification. The amplified cDNA was subjected to gel purification and cluster generation and then targeted for Illumina/Hiseq2500 sequencing. Reads from this sequencing with Phred scores greater than 35 were regarded as being of sufficiently high quality for evaluation.

### 2.7. Bioinformatics analysis of small RNA reads

We analyzed small RNA reads with CLC genomic workbench (https://digitalinsights.qiagen.com, CLC Bio, Qiagen, Germany) V12.0 and V22.0. We trimmed adapters, sorted reads and made annotations with Ensembl (Canis_familiris/canfam3.1.ncrna and Homo_sapiens/GRCh37.ncrna), and RNAcentral databases (https://www.rnacentral.org/). We used empirical analysis of differential gene expression (EDGE) to identify significant ncRNAs, based on FDR *p* value <0.05 and fold change > ±1.5.

### 2.8. qRT-PCR

A custom TaqMan gene expression assay was applied to determine the expression level of Y RNA (Thermo Fisher Scientific). qRT-PCR was performed as previously mentioned (Rahman et al. 2019). Briefly, total RNA was reverse transcribed to cDNA using the TaqMan MicroRNA Reverse Transcription Kit (Thermo Fisher Scientific) in accordance with the manufacturer’s protocol. A TaqMan First Advanced Master Mix Kit and a Quant Studio 3 real-time PCR system (Thermo Fisher Scientific) were used for qRT-PCR. All experiments were carried out in duplicate. The 2^−ΔΔCT^ method was used to evaluate the expression level. The internal controls used for the evaluation of relative expression were as follows: RNU6B for tumor tissue and cell lines, mir-16 for plasma, and mir-186 for EVs (Husna et al. 2021). The following TaqMan primer sequence was custom-designed for Y RNA fragments (Ensembl ID; ENSCAFT00000034244.1); 5′-GGCTGGTCCGAGTGCAGTGGTGCTTAC-3′.

### 2.9. Statistical analysis

Data were analyzed with the Mann–Whitney *U* test and an one-way analysis of variance (ANOVA) followed by the Kruskal–Wallis test to evaluate the relative expression of Y RNA. Receiver-operating characteristic (ROC) curves were plotted, and the relevant areas under the curve (AUCs) were determined using the Wilson/Brown method. All statistical analyses and graph visualization were performed with GraphPad Prism 9 (https://www.graphpad.com/).

## 3. Results

### 3.1. Clinical sample population

The clinical tissue samples in this study were obtained from 25 patients with COM, 17 of which had non-metastatic melanoma, and eight of which had metastatic melanoma. Plasma samples were obtained from 25 patients (a subset of the tissue sample population and only plasma samples were included), 15 of which had metastatic melanoma and 10 of which had non-metastatic melanoma. An overview of the patient information is summarized in Table 1.

**Table 1.** COM tissue and plasma sample information.

No.	Age (years)	Sex	Breed	WHO stage	Metastasis status	Tissue	Plasma
1	12.7	Male	Miniature	IV	P	P	P
2	14.8	Male	Mongrel	IV	P	P	P
3	10	Male	Golden Retriever	IV	–	P	P
4	10.11	Male	Miniature	I	–	P	–
5	7.11	Male	Miniature	I	–	P	P
6	10.9	Male	Miniature	IV	–	P	P
7	12	Male	Shiba	IV	P	P	P
8	13	Male	Pomerania	I	–	P	–
9	10.3	Male	Yorkshire	IV	–	P	P
10	10.2	Male	Chiwawa	IV	P	P	P
11	12.4	Female	Miniature	IV	–	P	P
12	14.6	Female	Miniature	II	P	P	P
13	15.2	Female	Mongrel	IV	–	P	–
14	12.11	Male	Miniature	IV	P	P	P
15	12.4	Male	Shiba	IV	P	P	P
16	15.2	Female	Mongrel	IV	P	P	P
17	10.8	Male	Miniature	IV	–	P	–
18	15.2	Male	Shiba	I	–	P	–
19	13.3	Male	Miniature	I	–	P	–
20	8.2	Female	Miniature	IV	–	P	P
21	12	Male	Mong	I	–	P	P
22	11.1	Male	Miniature	IV	–	P	P
23	15.6	Male	Pomeranian	II	–	P	P
24	15.3	Female	Mong	I	–	P	P
25	11	Male	Miniature	IV	–	P	–
26	15.3	Female	Mong	I	P	–	P
27	16.3	Male	Miniature	IV	P	–	P
28	11.8	Female	Miniature	I	P	–	P
29	14	Female	Dalmatian	II	P	–	P
30	12.1	Female	Toy poodle	IV	P	–	P
31	8	Male	Miniature	IV	P	–	P
32	10.1	Male	Mong	IV	P	–	P

P indicates “Present,” and (–) indicates “Absent.”

### 3.2. NGS profiling of ncRNAs

**Figure 1.**
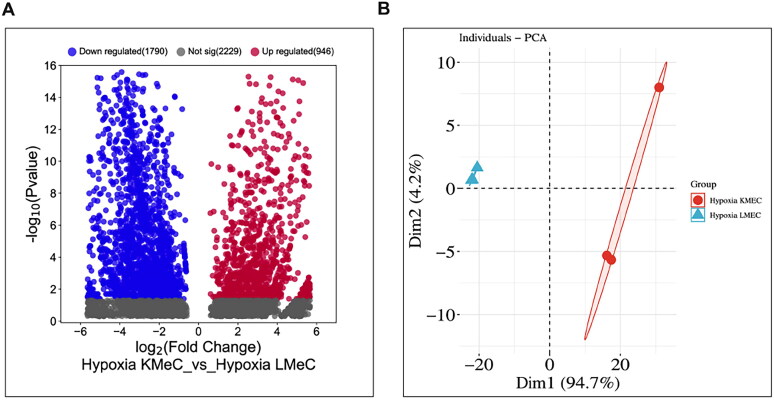
NGS profiling of ncRNAs in hypoxia KMeC and LMeC cell lines. **A**. Volcano plot showed differentially expressed upregulated and downregulated ncRNAs in hypoxic KMeC and LMeC cells. **B.** Principal component analysis (PCA) of hypoxic KMeC and LMeC cells.

We analyzed NGS reads obtained from KMeC (primary) and LMeC (metastatic) cells under hypoxic and normoxic conditions, targeting small ncRNAs other than miRNAs. We found that 1790 ncRNAs were downregulated and 946 ncRNAs were upregulated in hypoxic cells (Figure 1A). Among these ncRNAs, Y RNA (ENSCAFT00000034244.1) was significantly downregulated in hypoxic conditions in LMeC relative to KMeC cells (Table 2) and selected for further validation due to showing maximum mean value among these ncRNAs. A principal component analysis (PCA) plot demonstrated that the hypoxic KMeC cluster group differed from the hypoxic LMeC cluster group (Figure 1B). The sequence reads are available in the SRA repository (www.ncbi.nlm.nih.gov/sra, accession number: PRJNA629070).

**Table 2.** A partial list of hypoxia-related differentially expressed noncoding RNAs in between KMeC and LMeC cell lines (fold change (FC) > ±1.5, FDR *p* value < 0.05).

Name	RNA type	FC	FDR *p* value	Mean expression value	
				Hypoxia KMeC	Hypoxia LMeC
ENSCAFT00000034244.1	Y RNA	−1.96	0.02	170,478.67	96,964.33
ENSCAFT00000040529.2	SnoRNA	−1.92	0.04	6959.33	4052.33
ENSCAFT00000034287.1	snRNA	−1.89	0.00177	48,382.33	29,003.00
ENST00000415062.1	LncRNA	−46.25	0.00452	5.33	0
ENSCAFT00000091973.1	U4 RNA	−46.04	0.00423	5.33	0
ENSCAFT00000067406.1	U4-201 RNA	−46.02	0.00421	5.33	0
ENST00000577746.1	LncRNA	−44.62	5.23E–08	18	0.33
ENST00000619241.1	rRNA	−43.12	0.01	5	0
ENST00000428541.1	LncRNA	4.84	0.000126	5.67	31.67
ENST00000607541.1	LncRNA	4.85	0.00256	4	22.33
ENST00000671250.1	LncRNA	4.87	0.0000118	7.67	42.67
ENST00000547040.1	LncRNA	4.91	0.00845	2.33	13.33
ENST00000638592.1	LINC00174	5.56	0.00385	2.33	15.33
ENST00000436123.1	LINC00691	5.56	0.00538	2.33	15.33
ENST00000578492.5	LncRNA	5.7	0.01	2	13.67
ENSCAFT00000072615.1	LncRNA	5.88	0.05	2	13.67
ENSCAFT00000058714.2	LncRNA	5.91	0.000000042	10.67	72.67
ENST00000623717.1	LncRNA	6.93	0.000000851	4.67	37.33
ENSCAFT00000064745.1	LncRNA	7.82	7.82E–10	8	70.67
ENSCAFT00000076299.1	LncRNA	8.45	1.73E–09	19	186.33

### 3.3. qRT-PCR validation of Y RNA

#### 3.3.1. Relative expression in hypoxia and normoxia KMeC and LMeC cell lines

**Figure 2.**
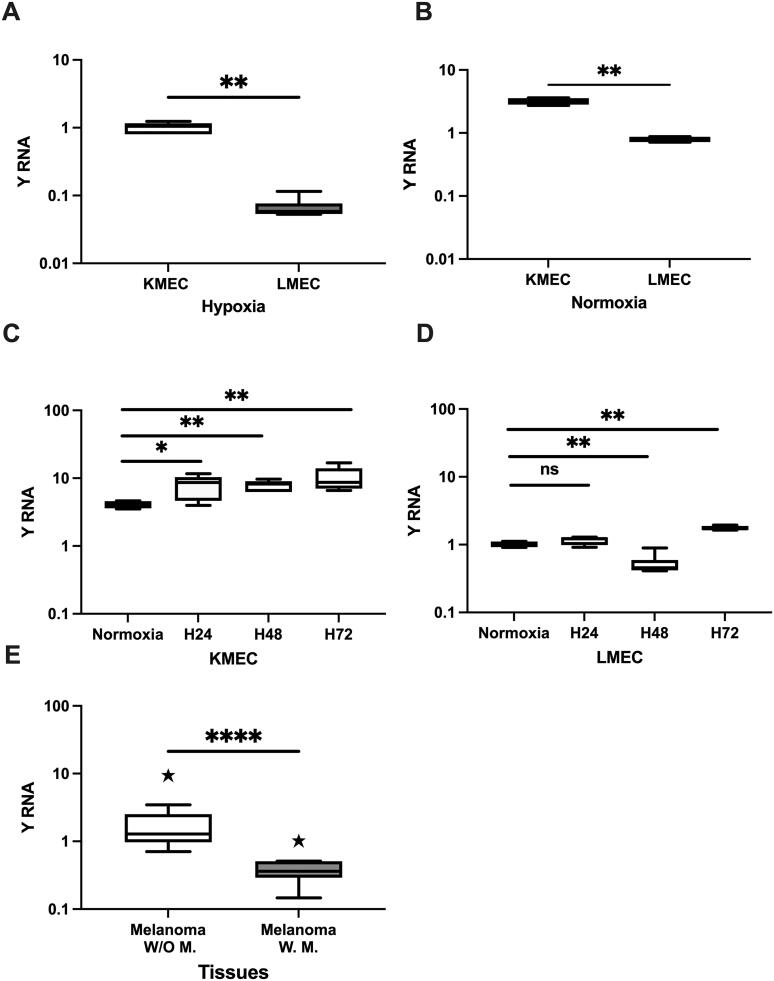
Relative expression of Y RNA in hypoxic and normoxic KMeC and LMeC cells and COM tissues by qRT-PCR. **A.** Relative expression in hypoxic KMeC and LMeC cells. **B.** Relative expression in normoxic KMeC and LMeC cells**. C,D.** Relative expression of Y RNA after different durations (24, 48, 72 h) in hypoxic KMeC cells versus normoxic cells. **E.** Relative expression level of Y RNA in non-metastatic melanoma (*n* = 17) and metastatic melanoma (*n* = 8) tissue samples. The *Y* axis indicates relative noncoding RNA expression levels in log10 units. KMeC and LMeC; replicate number = 6. Data were analyzed with one-way ANOVA (nonparametric) followed by the Kruskal–Wallis and Mann–Whitney *U* tests. Differences were considered significant when the *p* value was <0.05 (*p < 0.05, **p < 0.01, ****p < 0.0001). H: hypoxia; COM: canine oral melanoma; W/O: non-metastatic melanoma; W: metastatic melanoma, ns: not significant.

To validate the NGS results for Y RNA, we evaluated its expression in hypoxic KMeC and LMeC cells. We found that Y RNA was preferentially decreased in LMeC (*p* = 0.002, FC = 0.06) versus KMeC (Figure 2A) cells. We further investigated Y RNA relative expression under normoxic conditions. Under normoxia, Y RNA was also significantly decreased in LMeC (*p* = 0.002, FC = 0.24) versus KMeC cells, reflecting the trend observed under hypoxic conditions (Figure 2B). We then further investigated relationships between expression and a range of normoxic and hypoxic conditions. In KMeC cells, Y RNA was significantly increased (*p* = 0.008) for each of three different durations (24, 48 and 72 h) under hypoxia versus normoxia (Figure 2C). In LMeC cells, Y RNA showed no significant difference at 24 h, a significant decrease at 48 h (*p* = 0.002), and a significant increase (*p* = 0.009) at 72 h under hypoxia versus normoxia (Figure 2D). Overall, our data suggested that Y RNA was decreased in LMeC cells relative to KMeC cells under both hypoxic and normoxic conditions, and increased under hypoxic conditions relative to normoxic conditions.

#### 3.3.2. Relative expression in clinical tissue samples

We elucidated the expression pattern of Y RNA in COM tissue samples. Y RNA (*p* < 0.0001, FC = 0.21) was significantly decreased in the dogs with metastatic melanoma versus those with non-metastatic melanoma (Figure 2E). Y RNA was significantly increased in non-metastatic melanoma versus healthy controls (*p* < 0.001, FC = 6.90) (Suppl. Figure 1a). Collectively, Y RNA significantly distinguished metastatic melanoma from non-metastatic melanoma, which reflected the similar trend found in the hypoxic and normoxic cell line results.

#### 3.3.3. Relative expression in plasma and plasma-derived EV samples

**Figure 3.**
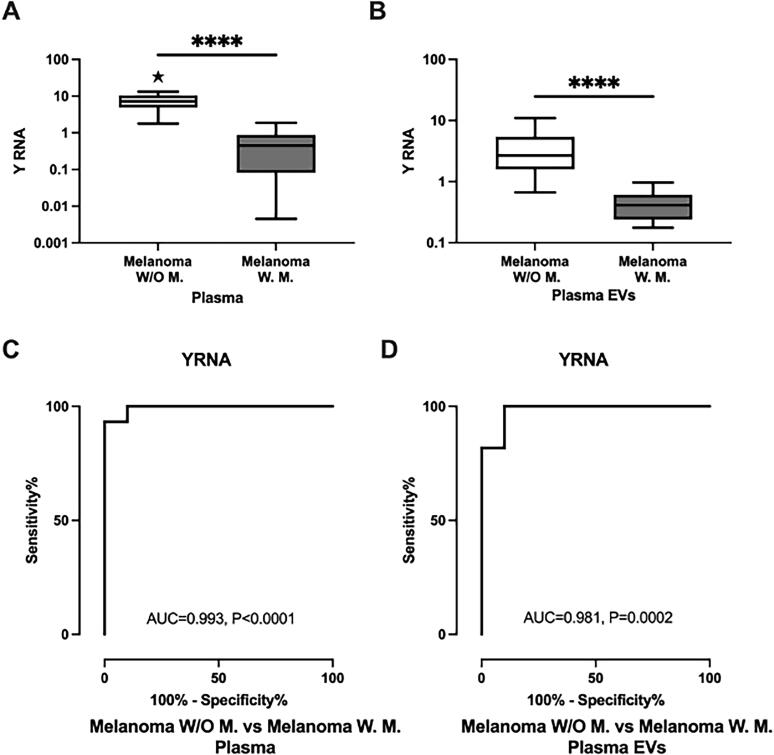
Relative expression of Y RNA in COM plasma and plasma-derived EV samples and its potentiality as a diagnostic biomarker. **A.** The relative expression level of Y RNA in non-metastatic melanoma (*n* = 10) and metastatic melanoma (*n* = 15) in plasma. **B.** The relative expression level of Y RNA in non-metastatic melanoma (*n* = 10) and metastatic melanoma (*n* = 11) in plasma-derived EVs. **C.** ROC curve (plasma) of the Y RNA for differentiating non-metastatic melanoma (*n* = 10) from metastatic melanoma (*n* = 15). **D.** ROC curve (plasma-derived EVs) of the Y RNA for differentiating non-metastatic melanoma (*n* = 10) from metastatic melanoma (*n* = 11). The *Y* axis indicated relative noncoding RNA expression levels in log10 units. Data were analyzed with a Student’s *t* test followed by a Mann–Whitney *U* test. Differences were considered significant when the *p* value was < 0.05 (****p < 0.0001). COM: canine oral melanoma; W/O: non-metastatic melanoma; W: metastatic melanoma; EVs: extracellular vesicles.

We further investigated Y RNA expression in plasma and plasma-derived EVs obtained from a subset of the population that provided COM tissue samples. Y RNA (*p* < 0.0001, FC = 0.06) was preferentially decreased in the dogs with metastatic melanoma versus those with non-metastatic melanoma (Figure 3A) in plasma. A similar trend in the relative expression of Y RNA (*p* < 0.0001, FC = 0.12) was found in plasma-derived EV samples (Figure 3B). In plasma and plasma EVs, Y RNA was significantly decreased in metastatic melanoma versus healthy controls (*p* = 0.0001, FC = 0.04 and *p* = 0.0001, FC = 0.34, Suppl. Figure 1b,c). Y RNA thus appeared to significantly differentiate metastatic melanoma from non-metastatic melanoma in plasma and EV samples. Furthermore, the expression pattern reflected the trends noted for hypoxic and normoxic cells and tissue samples from metastatic and non-metastatic COM patients.

### 3.4. Diagnostic profiles of Y RNA in COM

To evaluate the diagnostic value of Y RNA, we created ROC curves and evaluated the AUCs. The AUC of plasma Y RNA was 0.993 for dogs with metastatic melanoma versus those with non-metastatic melanoma (*p* < 0.0001; Figure 3C). We also assessed the diagnostic values of Y RNA in plasma-derived EVs, and in this analysis, the AUC of Y RNA was 0.981 for dogs with metastatic melanoma versus those with non-metastatic melanoma (*p* = 0.0002; Figure 3D). Overall, our data implies that Y RNA significantly discriminates metastatic from non-metastatic melanoma in both plasma and plasma-derived EVs.

## 4. Discussion

Dysregulated ncRNAs play a key role in tumor occurrence and progression and have a diagnostic or prognostic role in melanoma (Riefolo et al. 2019; Peng and Wang 2021). To our knowledge, this is the first report to consider whether hypoxically dysregulated Y RNA may play such roles in COM.

We initially identified downregulated Y RNA expression with hypoxia in the NGS analysis targeting ncRNAs in KMeC (primary) and LMeC (metastatic) cell lines. Therefore, we then went on to validate Y RNA expression in qRT-PCR analyses and evaluate it further using the samples from metastatic and non-metastatic COM patients. We found that the relative expression of Y RNA was significantly decreased in LMeC cells (a cell line of metastatic origin) relative to KMeC cells (a cell line of primary-site origin), under hypoxia, and we noted the same trend under normoxic conditions. However, the expression level of Y RNA in hypoxic KMeC cells was 2 to 2.5-fold higher than that in normoxic KMeC cells for each treatment duration evaluated (24, 48, and 72 h). Hypoxic LMeC cells showed Y RNA expression 1.5-fold higher than that in normoxic LMEC cells at 72 h, suggesting that Y RNA might be more resistant to stress under hypoxic than normoxic conditions. The expression level of Y RNA was significantly decreased in the tissue, plasma, and EVs from dogs with metastatic melanoma versus dogs with non-metastatic melanoma. ROC curve analysis demonstrated that the level of Y RNA could discriminate metastatic from non-metastatic melanoma, and thus has potential as a diagnostic biomarker.

Y RNAs are one of the least researched types of non-coding RNAs. They reportedly play a crucial role in initiating DNA replication, RNA stability, and cellular stress response (Gardiner et al. 2009; Kowalski and Krude 2015). Dysregulated Y RNAs and Y RNA-derived fragments (YRFs) may be involved in tumor carcinogenesis, and influence cell proliferation and inflammation (Guglas et al. 2020). Y RNAs are abundant in serum and plasma (Dhahbi et al. 2013; Solé et al. 2019). Y RNAs have potential diagnostic and prognostic significance in human prostate, bladder, and melanoma (Tolkach et al. 2017, 2018; Solé et al. 2019). Interestingly, in human oncology, Y RNAs and YRFs have been identified in various cancer malignancies and may be applied as cancer biomarkers (Dhahbi et al. 2013; Guglas et al. 2020). The three Y RNAs RNY3P1, RNY4P1, and RNY4P25 reportedly show significantly higher plasma levels in stage 0 melanoma patients than in healthy controls or patients with a more advanced stage of melanoma (Solé et al. 2019). A set of Y RNAs (RNY1, RNY3, RNY4, RNY5) was found to be downregulated in prostate and urinary bladder cancer (Tolkach et al. 2017, 2018). In human colon cancer, hY1 and hY3 RNA have been found to be upregulated versus healthy controls (Christov et al. 2008). A set of Y RNAs (hY1, hY3, and hY4) has reportedly increased in human cervix cancer patients (Christov et al. 2008). The presence of EV-derived dysregulated Y RNAs has also been reported in melanoma (Lunavat et al. 2015). Y RNAs have been featured in small-scale research with clinical samples in recent years; however, to our knowledge, no previous reports have identified alterations in Y RNA expression related to the response of tumors to hypoxia for any cancer.

Hypoxia, or low oxygen tension, plays a pivotal role in the tumor microenvironment and promotes metastatic progression (Rankin and Giaccia 2016). The functional roles of Y RNAs YRFs in hypoxic conditions remain unknown. Based on our results, we postulate that Y RNAs YRFs are more effective at resisting hypoxic cellular stress in the metastatic than the primary stage of melanoma, and our findings are potentially significant for understanding the response of the tumor microenvironment to hypoxia, and the role this plays in metastatic COM development. Overall, the downregulated hypoxia-related Y RNA expression trend in COM is consistent with the expression level of Y RNAs (RNY1, RNY3, RNY4, RNY5) in human melanoma, prostate, and urinary bladder cancer, and appears to distinguish the metastatic and non-metastatic melanoma.

Many researchers established that aberrations of the same genes are responsible for human and dog cancer (Rogers 2015). Thus, our findings on canine melanoma may be relevant to research on human melanoma, and the question of whether dogs could provide a model for the human disease.

The present study still has some limitations. First, the cohort sample was relatively small. We need further validation of the identified Y RNA in a large cohort sample to allow definite conclusions. Second, target mRNA genes, gene networks, and pathways associated with Y RNA need to be explored. Third, underlying molecular mechanisms of Y RNA need to be thoroughly investigated in a functional study.

Overall, we suggest that Y RNA may identify and distinguish metastatic from non-metastatic melanoma in dogs. Y RNA showed consistent trends in expression across hypoxic and normoxic cells and clinical melanoma samples. The expression level of Y RNA may be more resistant under the metastatic condition than at the primary state of melanoma, and thus be a potential biomarker for discriminating metastatic melanoma from melanoma without metastasis, paving the way for advances in disease tracking. However, more research will be crucial in fully elucidating the functional roles of Y RNA under hypoxia and normoxia, and the associated mRNA genes, and signaling pathways in COM disease development.

## Supplementary Material

Supplemental Material
