# Supplementary material for: Hypoxia-related Y RNA fragments as a novel potential biomarker for distinguishing metastatic oral melanoma from non-metastatic oral melanoma in dogs
Source: Vet Q. 2024 Jan 30;44(1):1–8. doi: 10.1080/01652176.2023.2300943 (PMC10829814; doi:10.1080/01652176.2023.2300943)
Supplement: Supplemental Material [file TVEQ_A_2300943_SM4908.docx]

**Supplementary Figure**





**Supplementary Figure (1 a-c)**: The relative expression of Y RNA in control vs Melanoma w/o metastasis, control vs Melanoma with metastasis, and Melanoma w/o metastasis vs Melanoma with metastasis. The Y-axis indicates relative noncoding RNA expression levels in log10 units. Data were analyzed with one-way ANOVA (nonparametric) followed by the Kruskal-Wallis and Mann-Whitney U tests. Differences were considered significant when the p-value was < 0.05 **p < 0.01, ***p< 0.001, ****p < 0.0001). COM: canine oral melanoma; W/O: non-metastatic melanoma; W: metastatic melanoma, ns: not significant.
